# During the COVID-19 Epidemic: Recommendations for the Admission and Treatment of Patients With Ovarian Cancer

**DOI:** 10.3389/fsurg.2021.740198

**Published:** 2021-11-18

**Authors:** Yu Ling, Ye Mingxia, Zhang Xiaoyan, Fan Yifan, Liu Peipei, Zhang Yue, Meng Yuanguang, Li Lian

**Affiliations:** ^1^Department of Obstetrics and Gynecology, The Seventh Medical Center, Chinese People's Liberation Army (PLA) General Hospital, Beijing, China; ^2^Medical School of Chinese People's Liberation Army (PLA), Beijing, China; ^3^School of Medicine, Nankai University, Tianjin, China; ^4^The Chinese People's Liberation Army (PLA) Hospital of the Ninth Eighth, Kaifeng, China

**Keywords:** the coronavirus disease 2019 (COVID-19), ovarian cancer, retrospective analysis, laparoscopic surgery, open surgery

## Abstract

**Background:** The coronavirus disease 2019 (COVID-19) had become a health care event endangering humans globally. It takes up a large number of healthcare resources. We studied the impact of COVID-19 on patients with ovarian cancer by comprehensively analyzing their admissions before and after the epidemic, and made reasonable suggestions to improve their current situation.

**Methods:** We randomly divided the enrolled patients into three groups, PreCOVID-19 Group (PCG) (2019.8.20–2020.1.20), COVID-19 Group (CG) (2020.1.21–2020.6.14), and Secondary Outbreak COVID-19 Group (SOCG) (2020.6.15–2020.10.10). One-way ANOVA and chi-square test were used for analysis.

**Results:** The number of patients from other provinces decreased significantly (*p* < 0.05). The total hospital stay during the epidemic was substantially more extended (*p* < 0.05). Before the epidemic, our department performed more open surgery while during the epidemic outbreak, we tended to choose laparoscopy (*p* < 0.01). We took a longer surgery time (*P* < 0.05). Patients had significantly less post-operative fever during the epidemic (*p* < 0.001).

**Conclusion:** During the COVID-19 epidemic, no patient was infected with COVID-19, and no patient experienced severe post-operative complications. We recommend maintaining the admissions of patients with ovarian cancer during the epidemic following the rules: 1. The outpatients must complete a nucleic acid test and chest CT in the outpatient clinic; 2. Maintain full daily disinfection of the ward and insist that health care workers disinfect their hands after contact with patients; 3. Increase the use of minimally invasive procedures, including laparoscopy and robotics; 4. Disinfect the ward twice a day with UV light and sodium hypochlorite disinfectant; 5. Patients need to undergo at least three nucleic acid tests before entering the operating room.

## Introduction

**Figure 1:**
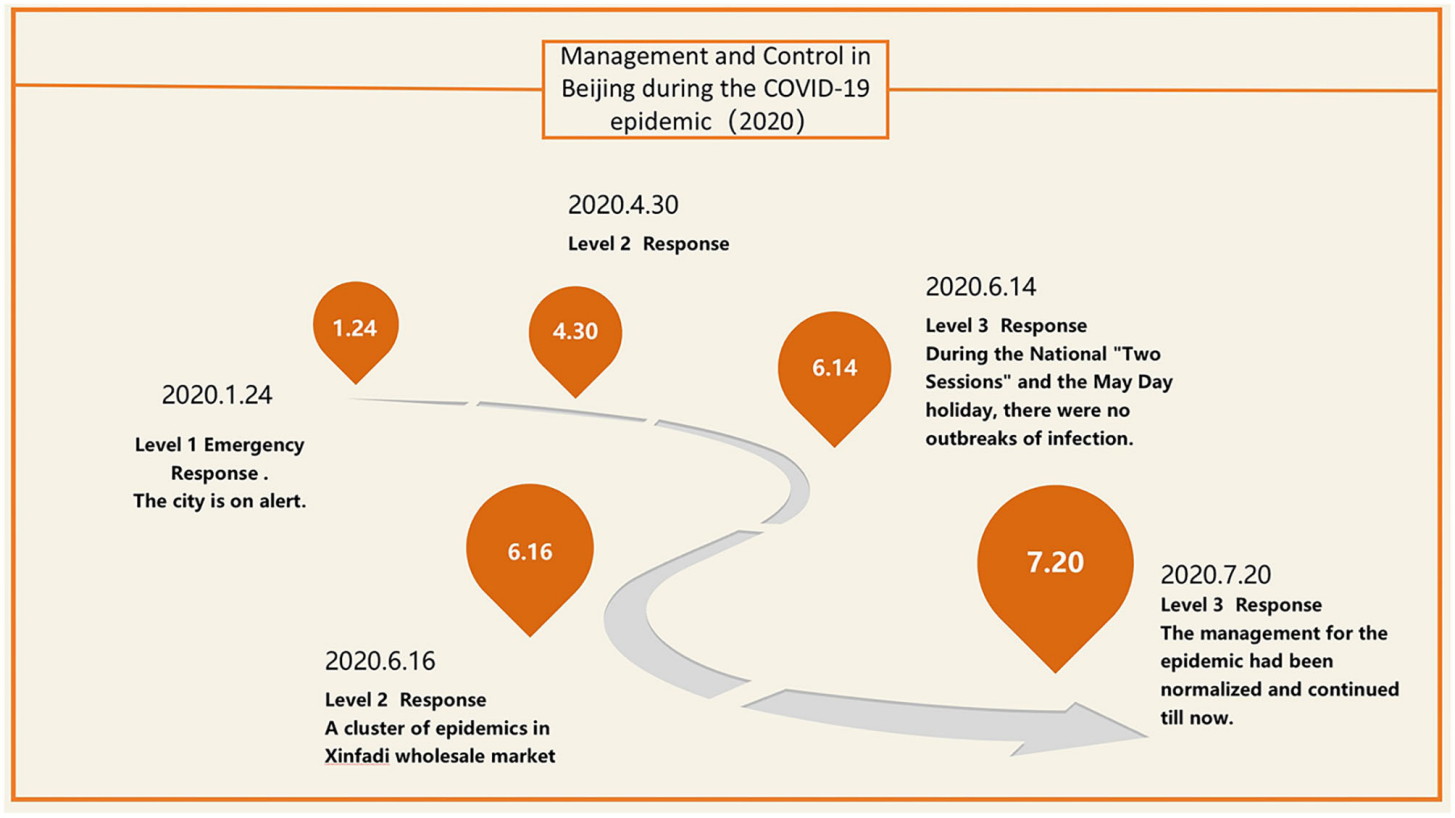
Management and control in Beijing during the coronavirus disease 2019 (COVID-19) epidemic (2020).

In December 2019, patients with novel coronavirus infection (COVID-19) appeared in Wuhan, and then COVID-19 spread across the country. Soon, the WHO declared COVID-19 a global infectious disease (1). Beijing is the capital of China, with a resident population of over 10 million and a large migrant population. When the epidemic spread to Beijing, there were many difficulties in its prevention and control (2). On January 24, 2020, the Beijing Municipal Government announced that it had entered the Level 1 Emergency Response. Following the regulations on the response level of COVID-19, our hospital has formulated relevant management measures, which mainly include: 1. All patients entering the outpatient building need to show their health codes and electronic travel cards (if there is a history of contact in the epidemic area, the health codes and travel cards will turn red); 2. Inpatients need to complete a nucleic acid screening in the outpatient clinic. After hospitalization, two more nucleic acid screening and chest CT examinations were performed, all of which were negative before the operation could be scheduled; 3. During the whole outpatient and hospitalization process, patients, nurses, doctors, and all personnel possibly related must wear masks. The ward must be disinfected every day, hands must be washed in time after contacting patients, and disinfectant must be applied. The above control measures continued until April 20, 2020. After that, the response was reduced to level 2, and our hospital adjusted the management to require it. Outpatients need to complete nucleic acid screening and chest CT in the outpatient department. The patients need one more nucleic acid screening after hospitalization. Surgeries can only be scheduled if all the above results are negative. After June 14, 2020, surgeries can be scheduled if patients have negative results from the nucleic acid screening and chest CT tests. However, this measure was only extended for 1 week. Due to the new outbreak in the XinFaDi Seafood wholesale market, the response level was adjusted to level 2 again (as shown in Figure 1). Our hospital used the control measures during the first outbreak again which lasted until July 30, 2020. With the Beijing Municipal Government reducing the response level to the third level again, our hospital began implementing the normalized epidemic management measures. The details were as follows: 1. All patients entering the outpatient building need to show their health codes and electronic travel cards; 2. Inpatients needed to complete a nucleic acid screening and chest CT examination in the outpatient clinic; if both resulted to negative, and the doctor scheduled the surgery; 3. During the whole outpatient and hospitalization process, patients, nurses, doctors, and all personnel possibly related must wear masks. The ward must be disinfected every day, hands must be washed in time after contact with patients, and disinfectant must be applied.

From January to October 30, 2020, our hospital achieved that none of the admitted patients had a nosocomial infection by implementing the above effective management measures. However, while preventing the spread of COVID-19, it may affect the diagnosis and treatment of gynecologic malignant tumors, especially ovarian cancer. Ovarian cancer is a common gynecologic malignancy and often progresses to an advanced stage at the visit of the patient. During the epidemic, the Beijing municipal government limited the access of patients and the time of pre-hospital examination was prolonged (3), which may delay the prognosis of ovarian cancer. In turn, it leads to more incredible surgical difficulty, more significant surgical comorbidities, greater blood loss, and greater expenses (4).

## Methods

### Study Design and Patients Collected

The Department of Obstetrics and Gynecology at the People's Liberation Army General Hospital has been consistently treating patients with gynecological cancer from all over the country during the COVID-19 epidemic. The first confirmed case of COVID-19 was announced in Beijing on January 20, 2020, after which the hospital began to implement strict management on outpatients and inpatients. This management continued until March 20, 2020, when there were no new confirmed cases in Beijing. On June 14, 2020, an outbreak of COVID-19 occurred in Beijing, and along with this, the management escalated again until August 6, 2020. Since then, the hospital management was normalized. Based on the above timeline, three phases were selected for the study, grouped as follows: a. August 20, 2019–January 19, 2020 [pre-COVID-19 group (PCG)]; b. January 20, 2020–June 14, 2020 [COVID-19 group (CG)]; c. June 15, 2020–October 10, 2020 [secondary outbreak COVID-19 group (SOCG)].

We enrolled 128 patients with ovarian cancer who received surgery at the gynecology department of the Chinese General Hospital of the People's Liberation Army (Beijing, China). All patients received cytoreductive surgery. Patients who received emergency surgical procedures were excluded. The operations were all performed by the same team of experienced and senior surgeons. According to the 2020 National Comprehensive Cancer Network (NCCN) clinical practice guidelines for ovarian cancer, all the patients were diagnosed based on pathological examination. The data which we used were integral and available. We informed the patients of the research methods and collected the data after the patients were discharged. The ethics committee of the Chinese General Hospital of the People's Liberation Army approved this study.

### Data Collection

Our research team obtained all the data from the electronic medical records. These data included: age, body mass index (BMI), comorbidity, pathological tumor, no, and metastases (TNM) classification, hemoglobin, CA125, the origin of patients (from Beijing or other areas), operative method (open surgery or laparoscopic surgery), operation time, blood loss, post-operative complications, post-operative fever, waiting time before hospitalization, and length of hospital stay. We defined the waiting time before hospitalization as the period before the patients came to our outpatient clinic for hospitalization.

### Statistical Analysis

GraphPad Prism 8 (GraphPad Software Inc., California, United States) was used for the statistical analysis. For the normal distribution, the data were expressed as mean ± SD (x ± s). We used the ordinary one-way ANOVA for data statistics. The comparison between the groups used Tukey's multiple comparisons test. A *P* < 0.05 was considered significant. The count data were expressed by frequency. The Chi-square test was used for the statistical analysis of the count data. A *P* < 0.05 was deemed to be significant.

## Results

### Study Population and Baseline Demographics

**Figure 2:**
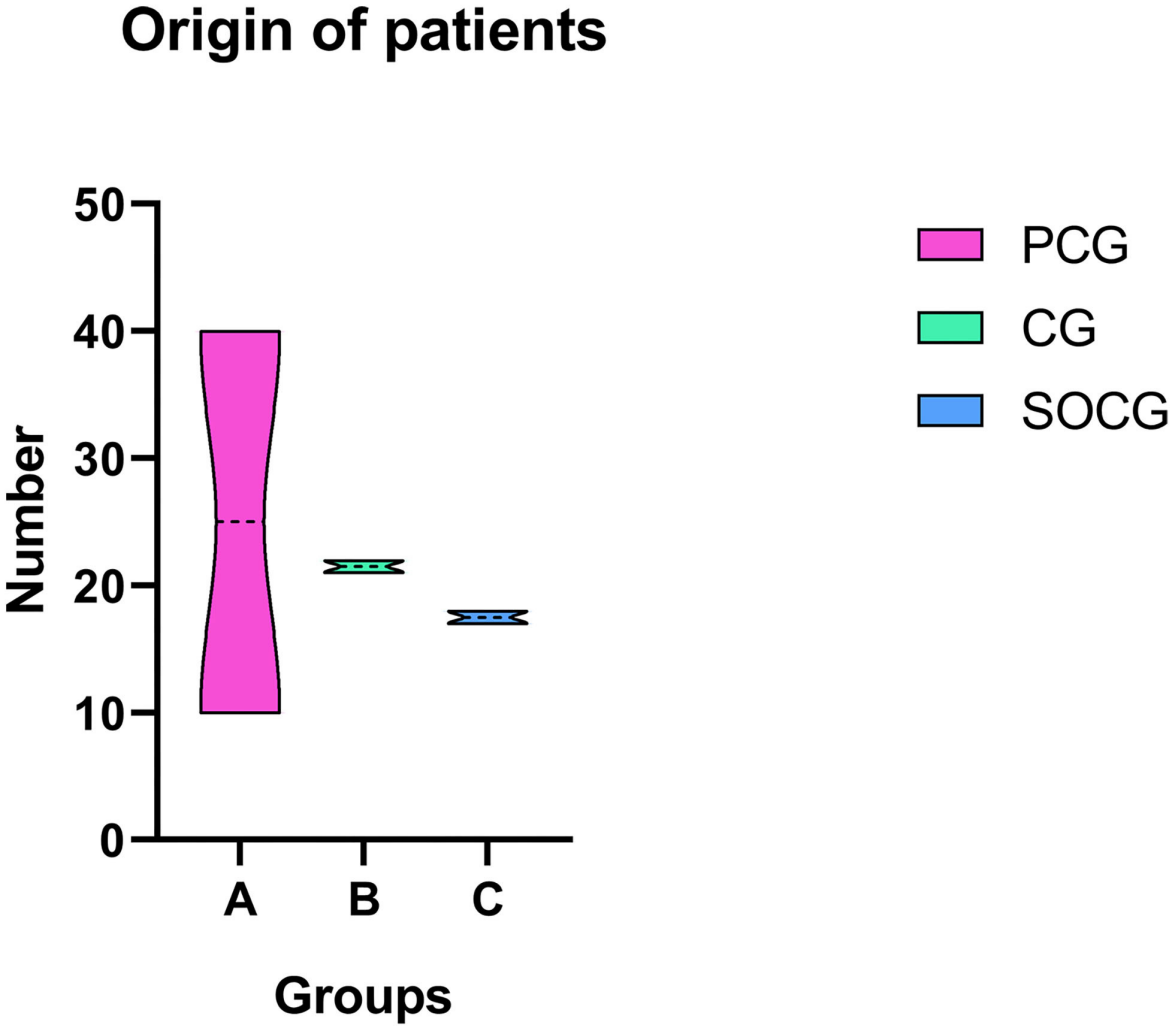
Origin of patients: We received more patients from other provinces in the COVID-19 period (*P* < 0.05). Pre-COVID-19 group-PCG (PCG): August 20, 2019–January 19, 2020; COVID-19 group-CG (CG): January 20, 2020–June 14, 2020; secondary outbreak COVID-19 group-SOCG (SOCG): June 15, 2020–October 10, 2020.

From August 20, 2019 to October 10, 2020, a total of 128 patients were enrolled. In this research, 49 patients were enrolled in the PCG group, 44 patients were enrolled in the CG group, while 35 patients were enrolled in the SOCG group. The patients hospitalized after January 20, 2020 were screened for symptoms of COVID-19 in the pre-operative period. All of them underwent a CT scan and the nucleic acid screening of COVID-19. All wore face masks during the hospital stay. The demographics of the patients are shown in Table 1 and Figure 2. There was no significant difference in age and body mass index among the three groups. We received more patients from other provinces in the COVID-19 period (*P* < 0.05).

**Table 1:** Baseline demographical data of all patients enrolled.

**Demographics**	**PCG (*N* = 49) M ± SD or *N* (%)**	**CG (*N* = 44) M ± SD or *N* (%)**	**SOCG (*N* = 35) M ± SD or *N* (%)**	***F***	***P***
Age(years)	54.3 ± 11.68	51.65 ± 11.4	53.89 ± 11.8	0.54	0.124
BMI	24.12 ± 4.085	23.32 ± 3.382	24.28 ± 4.001	0.72	0.4893
**Origin of patients**					0.0047^*^
Local patients	9	21	17		
Other provinces	40	23	18		

*PCG, Pre-COVID-19 group; CG, COVID-19 group; SOCG, second outbreak COVID-19 group; M, Mean; SD, Standard deviation; N, Number*;

* *p < 0.05, statistically different*.

### Statistics of the Accommodation and Treatment Environment of Patients

In the 5 months before the COVID-19 outbreak, our surgery team accommodated 49 patients with ovarian cancer. From January 20 to June 14, nearly 5 months, a total of 43 patients with ovarian cancer were accommodated. On average, our team admits only 8–9 patients per month. While the strict control measures lasted until May of this year, we admitted only five patients for the entire month of May and four patients in June. The length of hospital stay was significantly longer during the COVID-19 period (PCG:18.24 vs. CG:25, *P* < 0.01; PCG:18.24 vs. SOCG:22.8, *P* < 0.05), but there were no differences between CG and SOCG (CG:25 vs. SOCG:22.8, *P* = 0.496). During the second COVID-19 outbreak, our operative time was significantly longer than it was before the epidemic outbreak (PCG:240.9 vs. SOCG:298.4, *P* < 0.05). We used open surgery more often for ovarian cancer before the epidemic outbreak (*P* < 0.05). The probability of post-operative fever was significantly lower during the SOCG period (*P* < 0.0001).

**Figure 3:**
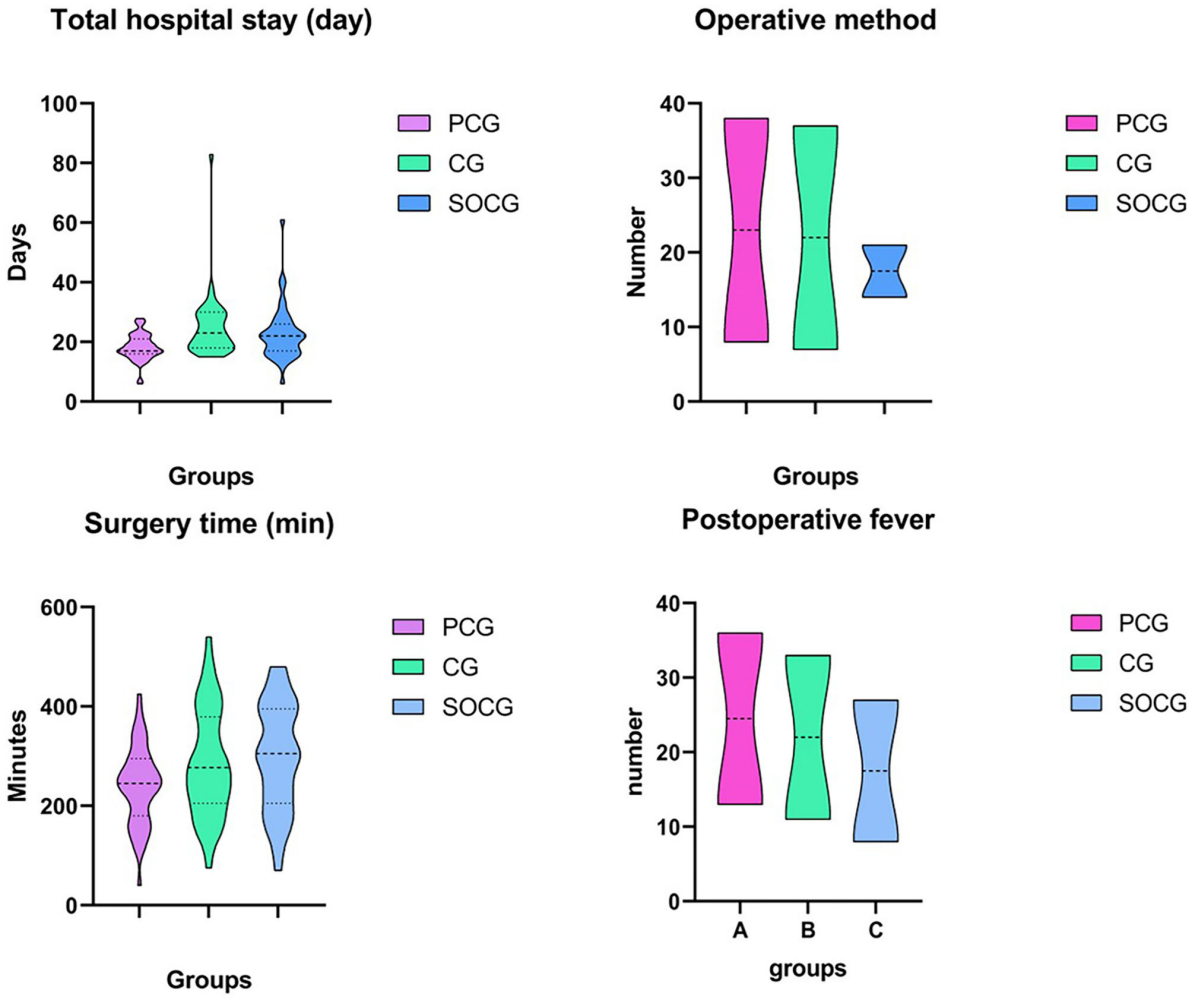
The number of patients from outside the province decreased significantly (*p* < 0.05). The total hospital stay during the epidemic was substantially more extended (*p* < 0.05). Before the epidemic, our department used more open surgery while after the epidemic outbreak, we tended to choose laparoscopy (*p* < 0.01), corresponding to the extended surgery time(*P* < 0.05). Patients had significantly less post-operative fever during the epidemic (*p* < 0.001). PCG: August 20, 2019–January 19, 2020; CG: January 20, 2020–June 14, 2020; SOCG: June 15, 2020–October 10, 2020.

There were no significant statistical differences in the waiting time before admission, Hemoglobin, CA125, clinical TNM staging, surgery-related complications, blood loss, transfusion of blood, lymph node metastasis, post-operative chemotherapy, and post-operative visit. These results are shown in Table 2 and Figure 3.

**Table 2:** Clinicopathological data of all patients enrolled.

**Clinicopathologic data**	**PCG (*N* = 49) M ± SD or *N***	**CG (*N* = 44) M ± SD or *N***	**SOCG (*N* = 35) M ± SD or *N***	***F***	***P***
Admission waiting (day)	16.36 ± 2.103	15.84 ± 2.982	9.686 ± 1.767	2.124	0.1412
Total hospital stay (day)	18.24 ± 0.637	25 ± 1.843	22.8 ± 1.475	7.116	0.0012^**^
Hemoglobin	111.4 ± 3.319	113.1 ± 2.760	117.8 ± 2.399	1.783	0.1724
CA125	512.6 ±136.8	384.4 ± 92.48	692.9 ± 252.4	0.765	0.4673
**Comorbidity**					0.2784
Yes	22	23	12		
No	27	21	23		
**Clinical TNM stage**					0.3029
I	6	5	6		
II	12	8	4		
III	21	20	22		
IV	10	11	3		
Surgery time (min)	240.9 ± 11.24	286.2 ± 16.47	298.4 ± 18.31	4.159	0.0178^*^
Blood loss (ml)	782.2 ± 187.0	580.7 ± 127.1	593.4 ± 121.4	0.558	0.5738
**Transfusion of blood (ml)**					0.3314
Yes	23	27	17		
No	26	17	18		
**Operative method**					0.0199^*^
Open	40	37	21		
Laparoscopic	9	7	14		
**Post-Operative fever**					0.0001^***^
Yes	36	33	8		
No	13	11	27		
**Lymph node metastasis**					0.0548
Yes	20	19	23		
No	29	25	12		
**Post-Operative chemotherapy**					0.1239
Yes	42	43	31		
No	7	1	4		
**Post-Operative visit**					0.1261
Yes	37	40	27		
No	12	4	8		

*PCG, Pre-COVID-19 group; CG, COVID-19 group; SOCG, second outbreak COVID-19 group; M, Mean; SD, Standard deviation; N, Number*;

* *p < 0.05*,

** *p < 0.01*,

*** *p < 0.001*.

## Discussion

Even during the worst period of the epidemic, our department maintained the admission of patients, especially those with gynecologic cancer. At the same time, we implemented strict control measures. But the number and composition of diseases that admitted patients had changed markedly. The proportion of patients with ovarian cancer admitted to our department rose by 20% during the CG and SOCG periods, with a significant increase in the local patients admitted; both could be attributed to travel restrictions and blockades in Beijing (3).

Ramirez et al. suggested that neoadjuvant chemotherapy should be considered to downplay the condition for patients with advanced ovarian cancer. When the epidemic was uneventful, these patients should consider surgery (5, 6). Kobayashi et al. (7) supported the above management recommendations, concluding that pre-operative chemotherapy can effectively alleviate the condition and not affect the prognosis of the patients (8). But subsequently, a study from Turkey showed that gynecological cancer surgery should continue during the COVID-19 pandemic while adhering to prevention and control measures. The surgeon should only consider patients with documented COVID-19 infection non-surgical treatment (9). Our retrospective analysis gave the same suggestion as the study from Turkey.

We found that the total hospital stay during the epidemic was substantially more extended (*p* < 0.05). Because after being hospitalized, another nucleic acid test was needed. Patients would be isolated in a separate ward for 3 days. If the nucleic acid result were negative, we would include the patient in the surgical range. If the patient had a negative nucleic acid result but an abnormal chest CT, a consultation with the respiratory medicine department is also required to rule out COVID-19. Patients need to undergo at least three nucleic acid tests before entering the operating room. The above reasons all prolonged the total hospital stay.

Before the epidemic, our department used more open surgery while after the epidemic outbreak, we tended to choose laparoscopy (*p* < 0.01). corresponding to the extended surgery time (*P* < 0.05). Although previously shown, laparoscopic surgery was not associated with a significant difference in operative time than open surgery (10, 11). However, data from this study suggest that surgical time was prolonged during the epidemic. We performed more meticulous laparoscopic operations during the outbreak to reduce the intraoperative blood loss of the patients and prevent post-operative fever. This is probably responsible for the longer surgery time.

We performed more laparoscopic surgeries, which effectively reduced blood loss to the extent that it prevented the occurrence of post-operative fever (*P* < 0.001).

The current study focused on how to treat patients infected with COVID-19 and what complications follow the treatment (12, 13). Few studies have focused on patients with ovarian cancer not infected with COVID-19 during the epidemic. Monk et al. showed that the outpatient visit time and the hospitalization time of patients should be reduced (14). It may prevent the spread of the COVID-19. But the diagnosis and treatment of patients with ovarian cancer is time-consuming and reducing the time might reduce the treatment, which has the potential to result in a suboptimal treatment effect (15). How should this part of patients be managed, treated to prolong survival, and guarantee patient safety?

We achieved sustained access to patients with cancer during the epidemic without a single case of nosocomial infection and a single post-operative adverse event because of strict and effective management measures. We recommend maintaining regular admissions of patients with ovarian cancer during the epidemic. The prerequisite is that patients must complete one nucleic acid test and one chest CT in the outpatient clinic with negative results. Maintain full daily disinfection of the ward by disinfecting the ward twice a day with UV light and sodium hypochlorite disinfectant, and insist that health care workers disinfect their hands after contact with patients. Increase the use of minimally invasive procedures, including laparoscopy and robotics. Patients need to undergo at least three nucleic acid tests before entering the operating room.

## Limitations

This study is a single-center retrospective study with an insufficient number of cases, which should be expanded to carry out multicenter studies. The sample size was large enough to allow for more generally accurate guidance.

## Data Availability Statement

The original contributions presented in the study are included in the article/supplementary material, further inquiries can be directed to the corresponding author/s.

## Author Contributions

All authors participated in writing the article.

## Funding

The medical big data research and development of Chinese People's Liberation Army General Hospital: Ovarian Cancer Precision Treatment Database Construction, 2018MBD-021.

## Conflict of Interest

The authors declare that the research was conducted in the absence of any commercial or financial relationships that could be construed as a potential conflict of interest.

## Publisher's Note

All claims expressed in this article are solely those of the authors and do not necessarily represent those of their affiliated organizations, or those of the publisher, the editors and the reviewers. Any product that may be evaluated in this article, or claim that may be made by its manufacturer, is not guaranteed or endorsed by the publisher.
